# Production of Green Diesel via the Ni/Al Mo Hydrotalcite Catalyzed Deoxygenation of Rapeseed Oil

**DOI:** 10.3390/molecules30081699

**Published:** 2025-04-10

**Authors:** Giuseppe Di Vito Nolfi, Katia Gallucci, Vittoria Mucciante, Leucio Rossi

**Affiliations:** 1 Department of Physical and Chemical Sciences, University of L’Aquila, 67100 L’Aquila, Italy; giuseppe.divitonolfi@graduate.univaq.it (G.D.V.N.); vittoria.mucciante@univaq.it (V.M.); 2 Department of Industrial and Information Engineering and Economics, University of L’Aquila, 67100 L’Aquila, Italy; katia.gallucci@univaq.it

**Keywords:** green diesel, hydrotalcites, deoxygenation reaction, heterogeneous catalysis

## Abstract

Growing concern about anthropogenic climate change and the continuous increase in the energy demand have driven the need to explore new energy sources, particularly in the transportation sector. Biodiesel is one of the most widely used biofuels, but its disadvantages restrict its use in blends with conventional diesel. A better alternative is green diesel, a hydrocarbon biofuel that can be used in its pure form and is produced through the catalytic deoxygenation of vegetable oils. In this study, a NiMoAl catalyst derived from layered double hydroxides (LDHs) was synthesized and used for the catalytic deoxygenation of rapeseed oil to produce green diesel. The catalyst was characterized using IR, XRD, and BET analysis. The reactions were carried out in a batch reactor, and parameters such as the temperature, pressure, catalyst loading, and reaction time were examined. The results demonstrated that the complete conversion of rapeseed oil was achieved under optimal conditions (320 °C, 40 bar H_2_, 4 wt% catalyst), with a diesel-range hydrocarbon content of over 90%. The recyclability of the catalyst was also evaluated, showing sustained activity over multiple reaction cycles while maintaining high conversion and selectivity toward hydrocarbons in the diesel range.

## 1. Introduction

**Figure 1:**
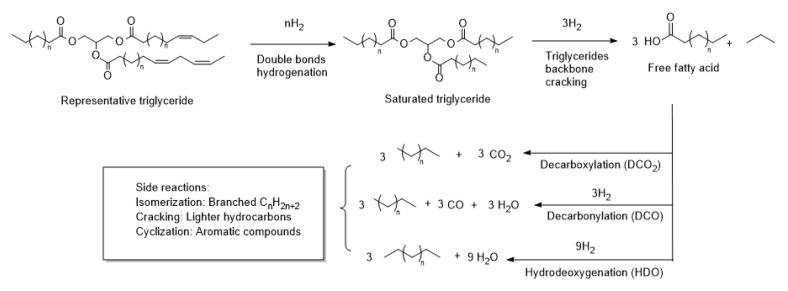
CDO reaction.

Nowadays, the development of renewable and ecofriendly energy sources has become crucial due to the exploitation of fossil fuels and the global warming associated with their combustion. In addition, constant economic growth increases energy demand to the extent that global energy consumption is projected to rise by approximately 1.1% per year from 2022 to 2050. As a result, fuel demand in the transport sector is predicted to increase by between 8% and 41% from 2022 to 2050 [1]. The development of biofuels may provide a viable solution to the problem of how to satisfy this increased energy demand while mitigating environmental pollution. Biofuels can be produced from biomass, representing the main renewable energy source for solid, liquid, and gaseous biofuels [2]. An example of biomass is vegetable oils, which are an ideal feedstock for biofuel production because they have similar physicochemical properties to petroleum derivatives, such as high energy density and similar chemical composition [3]. Furthermore, plant-derived biofuels can significantly reduce greenhouse gas emissions because they are considered CO_2_ neutral, with lower SO_x_ emissions due to the low sulfur content of plants [4,5]. A widely used biofuel is biodiesel, which can be obtained through the transesterification of vegetable oils. However, biodiesel is not an effective alternative to conventional diesel due to several drawbacks, including its limited miscibility with petroleum diesel, high oxygen content, high corrosivity, and low storage stability [6]. In contrast, green diesel, also derived from vegetable oils, is a hydrocarbon biofuel, mainly composed of *n*-C_15_-C_18_ alkanes (as vegetable oils mainly consist of C_15_-C_18_ fatty acid) that are fully miscible with petroleum diesel and have zero oxygen content, high storage stability, and high heating value [7,8]. Green diesel can be produced through the catalytic deoxygenation reaction (CDO) of vegetable oil, in which the triglycerides (TGs) are thermally treated, typically in a hydrogen atmosphere with heterogeneous catalysts, and converted into hydrocarbons. Briefly, the double bonds of the triglycerides are first hydrogenated, and then the triglyceride is broken to form free fatty acids and propane; the most common mechanism for triglyceride cleavage is β-elimination [9,10,11]. Finally, hydrocarbons are formed through three main reactions: hydrodeoxygenation (HDO), in which fatty acid oxygen functionality is removed as H_2_O to produce hydrocarbons with the same carbon number of the starting fatty acid; decarbonylation (DCO), and decarboxylation (DCO_2_), which produce hydrocarbons with one fewer carbon atom than the starting fatty acid and the oxygen is removed as CO (DCO) or CO_2_ (DCO_2_) (Figure 1) [12]. The reaction mechanism has already been discussed in numerous studies and reviews. It involves successive hydrogenation and hydrogenolysis reactions to produce several intermediates, such as aldehydes, ketones, alcohols, and esters of fatty acids. A more in-depth and explicative discussion of the reaction mechanism is reported in two reviews [12,13]. Several side reactions can occur during this process, such as cracking (whereby hydrocarbons and fatty acids are cleaved to form short-chain hydrocarbons), oligomerization via a Lewis acid-catalyzed Diels–Alder reaction (resulting in coke and heavy hydrocarbons formation) and gas-phase reactions such as water gas shifts [14]. Many factors influence all the reactions involved in this process, including temperature, hydrogen partial pressure, and, most importantly, the type of catalyst used [15].

Sulfided transition metal catalysts are widely used in CDO processes as they are highly active [16,17,18,19,20,21]. However, their applicability is limited by the rapid deactivation caused by sulfur leaching. The use of sulfiding agents can mitigated this drawback, but this approach leads to sulfur contamination of the biofuels produced [22,23]. Noble metal catalysts were also found to be highly active towards CDO [24,25,26,27,28]. While these catalysts overcome the limitations of sulfided catalysts, their high cost remains a significant disadvantage. An appropriate amount of nickel provides a more active catalyst than Pd and Pt catalysts [29], and bimetallic catalysts are more efficient than noble metal catalysts [30]. Reduced-state transition metal catalysts are also a suitable alternative to sulfided catalysts, offering comparable activity with significantly higher stability [31]. Typically, these catalysts are supported metal catalysts in which the metals (Ni, Co, Mo, W, Zn, Fe) are supported on different supports (Al_2_O_3_, SiO_2_, ZrO_2_, composite, zeolites, carbon materials) [32,33,34,35,36,37,38,39]. A further category of transition-metal-based catalysts comprises nitride and carbide catalysts. As in the case of transition-metal-based catalysts in the reduced state, these catalysts (typically supported Mo_2_C, W_2_C, NiMo_2_C, Mo_2_N, VN, NiMo_2_N, and WN) prove to be a cost-effective alternative to noble metals by showing comparable catalytic activities. Moreover, they present greater stability than sulfide catalysts but require severe synthesis conditions (typically 700 °C) [40,41,42,43,44,45]. Layered double hydroxide (LDH)-based catalysts constitute an important class of transition-metal-based catalysts. They can be simply synthesized by co-precipitation methods. These materials are anionic clay with a layered structure and a general formula (M(II)_1−x_M(III)_x_(OH)_2_]^x+^(A^n−^_x/n_)·mH_2_O, where M(II) and M(III) are the bivalent and trivalent metals, respectively, A is the counterion balancing the positive charge of the layers, and x = M(III)/(M(II) + M(III)) [46]. LDHs are highly tunable materials, as their properties can be adjusted by altering the divalent or trivalent cations, their molar ratios, or the counterions. Upon calcination, LDHs are converted into mixed metal oxides with excellent catalytic properties [47]. LDH-derived catalysts have been widely used for the hydrodeoxygenation of substates other than vegetable oils, such as vanillin, guaiacol, and lignin [48,49,50,51,52,53] and model compounds of vegetable oils [54,55,56,57]. To the best of our knowledge, LDH activity has been less thoroughly explored in the catalytic deoxygenation of real feedstock [58,59]. The primary objective of this study is to repurpose and evaluate, for the first time, the catalytic activity of the NiMoAl catalyst developed by Arias et al. [60,61] in the catalytic deoxygenation of rapeseed oil. The primary goal of the study is to assess the effectiveness of the NiMoAl catalyst in green diesel production, optimizing reaction conditions to maximize the diesel yield while minimizing the temperature, pressure, catalyst loading, and reduction time—key factors for industrial cost efficiency. Catalyst stability was also investigated, as long-term durability reduces the need for regeneration. Finally, we explored its use without a pre-reduction step, achieving significant time and cost savings without compromising performance.

## 2. Results

### 2.1. Catalyst Synthesis

A NiMoAl layered double hydroxide was synthesized using the well-known coprecipitation method proposed by Arias et al., starting with solutions of Ni(NO_3_)_2_·6H_2_O and Al(NO_3_)_3_·9H_2_O and terephthalic acid as a precursor for the counteranion spacer [60,61]. Molybdenum was inserted in the LDH structure (in this state, the LDH is named NiAl LDH) by substituting the counteranion with (NH_4_)_6_Mo_7_O_24_·4H_2_O [61]. After Mo insertion, the solid is referred to as NiMoAl LDH. The resulting NiMoAl LDH was then calcined to produce mixed metal oxides (namely, NiMoAl C., where C. means calcined). The catalyst was tested in its reduced form (NiMoAl R., where R means reduced) under various reaction conditions to determine the optimal parameters, and a study of the reusability of the catalyst was also carried out. Additionally, the catalytic activity of the calcined form of the catalyst (i.e., without the pre-reduction step) was also evaluated.

### 2.2. Catalyst Characterization

The catalyst was characterized to evaluate the effectiveness of the synthesis of the NiMoAl catalyst compared to the analogous one synthesized by Arias et al. [60,61], thereby confirming its textural properties. The catalyst’s properties were analyzed using the FT-IR, XRD, ICP-MS, and BET-BJH techniques.

**Figure 2:**
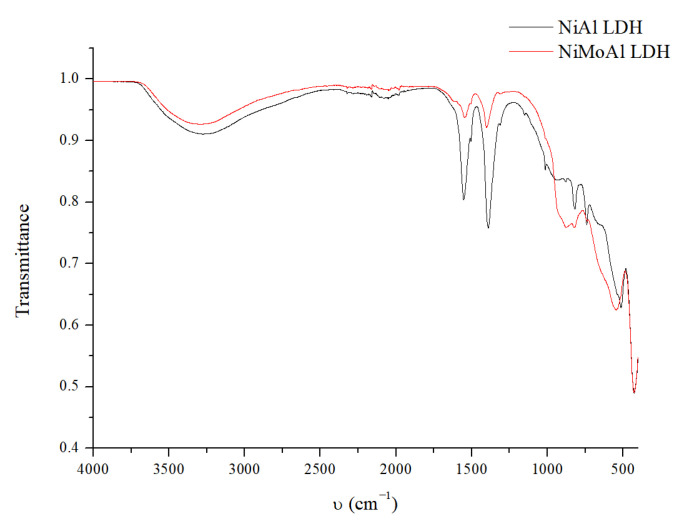
IR spectra of the NiAl LDH uncalcined precursor (black line) and uncalcined NiMoAl LDH (red line).

The IR spectra of the NiAl LDH precursor (the red line in Figure 2) and the NiMoAl LDH are shown in Figure 2.

The IR spectra are consistent with those reported by Arias et al. for these materials [60]. The broad band between 3600 and 3200 cm^−1^ is characteristic of structural OH and water molecules within the hydrotalcite layers. The characteristic peaks corresponding to the vibrational modes of the terephthalate carboxylates (symmetric and antisymmetric stretching) were observed at 1552 and 1389 cm^−1^, respectively. The signal at 430 cm^−1^ is assigned to the Ni-O bond vibration, while the band centered at 513 cm^−1^ is associated with Al-O stretching. After Mo intercalation, the IR spectrum (red line Figure 1) shows a significant decrease in the intensity of the terephthalate stretching signals, confirming the incorporation of heptamolybdate into the structure. This is further supported by two bands centered at around 900 cm^−1^ and 640 cm^−1^. The band near 940 cm^−1^ corresponds to Mo=O bond stretching at terminal units, while the bands near 885 cm^−1^ and 645 cm^−1^ correspond to the antisymmetric vibration modes of Mo-O-Mo units at the corners and edges of the MoO_6_ octahedron, respectively. These findings confirm that the molybdenum species in the NiMoAl LDH catalyst are octahedrally coordinated with oxygen through heptamolybdate incorporation during the ion exchange process [61].

**Figure 3:**
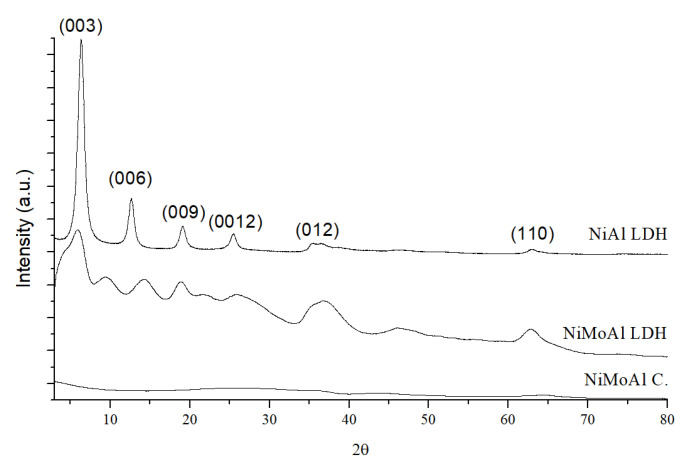
XRD plot of the various states of the synthesized material.

Figure 3 shows the XRD diffractograms of the uncalcined NiAl LDH and NiMoAl LDH and calcined NiMoAl C.

The XRD diffractograms confirm the effectiveness of the synthesis. As observed by Arias et al. [60], the NiAl LDH precursor diffractogram exhibits the characteristic diffraction pattern of LDHs, i.e., weak and broadened reflections at angles 2θ > 30° and more intense and sharp reflections at lower angles (2θ < 30). In addition, the (003) peak confirms the formation of an LDH with terephthalate as the intercalating ion [42]. After the ion exchange, the material (NiMoAl LDH) significantly loses its crystallinity. However, it retains some of the characteristic reflections of the NiAl LDH precursor, including the (003), (009), and (0012) peaks and the reflections at angles greater than 35°. In addition, the diffractogram of uncalcined NiMoAl shows three new peaks at 9.4°, 14.8°, and 22.0°, which are attributable to the intercalation of the heptamolybdate ion as suggested by Arias et al. [61]. After calcination, the resulting material (NiMoAl C.) becomes completely amorphous. This observation is supported by the work of Li et al. [62]. The authors show the XRD patterns of a similar NiMoAl LDH calcined at different temperatures, confirming that no crystalline phases are detectable at calcination temperatures comparable to those used in our work.

The metal content of the catalyst was evaluated via the ICP-MS analysis of the calcined catalyst (NiMoAl C.). The results are reported in Table 1.

The Ni/Al molar ratio obtained from the ICP-MS analysis perfectly matches the nominal Ni/Al molar ratio chosen for the catalyst synthesis. The ICP-MS analysis also confirms the effective Mo intercalation in the LDH precursor after the ion exchange. However, the experimental Ni/Mo molar ratio is lower than the theoretical ones, suggesting that not all the heptamolybdate was intercalated within the LDH structure. This interpretation is supported by IR spectra that still reveal the presence of residual terephthalate carbonyl stretching bands. Similar findings were reported by Arias et al., who also observed that the Mo intercalation remained incomplete [61]. They attributed this behavior to the persistence of the residual terephthalate and a possible co-exchange of a carbonate anion with a heptamolybdate anion.

**Figure 4:**
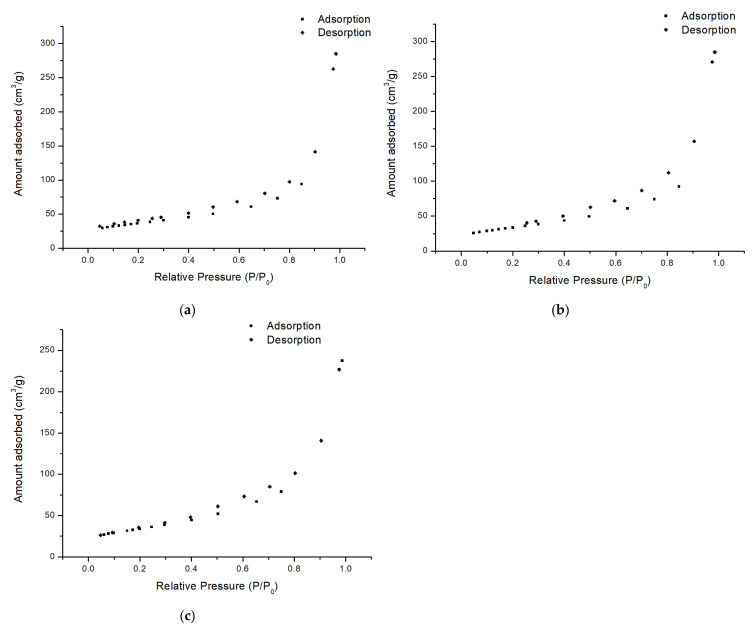
BET isotherms of (**a**) NiAl LDH (uncalcined precursor), (**b**) NiMoAl C., and (**c**) NiMoAl R.

The BET isotherms of the NiAl LDH precursor, NiMoAl C., and NiMoAl R. are shown in Figure 4. The analysis of the reduced sample is included because the adsorptive capacity of the reduced sample governs the adsorption of hydrogen in the reaction environment.

In all cases, the curves are type IV, which is typical for mesoporous material (pores with a diameter between 2 and 50 nm), accompanied by hysteresis when the pore width exceeds a certain critical width (e.g., for nitrogen adsorption in cylindrical pores at 77 K, hysteresis starts to occur for pores wider than ~4 nm). Furthermore, the hysteresis-type H3 loop was recognized, indicating non-rigid aggregates of plate-like particles (e.g., certain clays) or pore networks consisting of macropores that are not completely filled [62,63]. The mesoporous structure is further confirmed by the average pore diameter reported in Table 2. In all cases, a pore diameter in the range of 12–14 nm is calculated. Given the high metal content of these materials, the measured surface area is large and is in agreement with the results that Arias et al. observed for these systems [64].

### 2.3. Catalytic Deoxygenation Reaction of Rapeseed Oil

The catalytic activity of the catalyst was evaluated for the synthesis of green diesel using rapeseed oil as the feedstock. The reactions were carried out in a batch reactor by varying the reaction conditions, including temperature, pressure, and catalyst/oil ratio.

A first set of experiments was carried out with 2.00 g of rapeseed oil and 20.0 g of hexane (oil to solvent ratio 1:10), maintaining a fixed reaction time of 6.0 h. The temperature, H_2_ pressure, and catalyst/oil wt% ratio were set as follows: T = 320 °C, 240 °C, P(H_2_) = 20 bar, 40 bar, catalyst/oil wt% ratio = 10, 4. Additionally, a reaction under intermediate reaction conditions was carried out (280 °C; 30 bar H_2_ pressure; 7.0 wt% catalyst). Prior to the reactions, the catalyst was reduced in situ at 320 °C, 60 bar H_2_, and 8 h of reduction time. Following this initial screening, the catalyst’s activity was further investigated by varying the reaction time (6 h, 4 h, and 2 h) and catalyst reduction time (8 h, 4 h, and 2 h). Once the best reaction conditions had been identified, a recyclability study of the catalyst was performed. Finally, the catalyst’s activity was also tested without the pre-reduction step. At the end of each reaction, the catalyst was filtered, and the reaction product was recovered to assess the OLP yield (OLP = organic liquid product). The OLP yield was calculated by dividing the weight of the recovered crude reaction mixture (gOLP) by the weight of the starting oil (gOIL). The obtained product was analyzed using FT-IR, GC-FID and GC-MS. To verify the presence of unreacted oil (triglycerides and free fatty acids), an aliquot of OLP was subjected to transesterification according to the standard procedure AOAC 969.33 [65]. The resulting FAME (fatty acid methyl ester) content was determined via GC analysis by integrating the chromatographic peak areas and calculating the area percentage ratio.

For each reaction, the reaction conversion (χ), the OLP yield (OLP (%)), the green diesel yield (GD %), and the selectivity (S) were reported [66,67]. Additionally, the distribution of reaction products was analyzed, including the percentage of olefins, linear C_8_-C_14_ alkanes, linear C_15_-C_18_ alkanes, linear alkanes with more than 18 carbon atoms, branched hydrocarbons, and free fatty acid and triglycerides (as FAME).

The conversion (χ) was calculated using the formula reported in Equation (1), where FAME is the sum of the area % of the chromatographic peaks corresponding to FAME, gOLP refers to the weight (in grams) of the recovered crude reaction mixture, and gOil is the grams of the weighted oil:

(1) $$\chi =1-\left(\frac{FAME\times gOLP}{gOil}\right)\times 100$$

The green diesel yield (GD %) was calculated as reported in Equation (2), where *n*-C_15_-C_18_ represents the sum of the area percentage of the chromatographic peaks in the range C_15_-C_18_:

(2) $$GD\left(\%\right)=\frac{n–C_{15}–C_{18}\times gOLP}{gOIL}\times 100$$

Selectivity in the CDO reaction reflects the catalyst’s preference for a specific reaction pathway (see Figure 1) and was determined as the ratio between the sum of the area of C_16_ and C_18_ peaks and the sum of the area of C_15_ and C_17_ peaks, according to Equation (3):

(3) $$S=\frac{C_{16}+C_{18}}{C_{15}+C_{17}}\times 100$$

A selectivity value > 1 indicates a preference for the HDO pathway because more C_16_ and C_18_ are produced (formed by HDO pathways) than C_15_-C_18_ (derived from DCO/DCO_2_). Thus, the value of the fraction present in Equation (3) will be greater than 1. Similarly, a value < 1 suggests the dominance of the DCO/DCO_2_ pathway. The choice between HDO and DCO/DCO_2_ in green diesel production involves a trade-off between costs and fuel quality. The HDO pathway requires high hydrogen consumption, leading to increased costs due to H_2_ storage and transportation [68]. On the other hand, the HDO route produces fuel with higher atom economy, heating values, and cetane numbers, with water as the only byproduct. In contrast, DCO/DCO_2_ reduces hydrogen consumption and can operate at low pressures or in an inert atmosphere, lowering costs [69]. However, it leads to the loss of a carbon atom, decreasing the cetane number and heating value of the biofuel. It also generates CO and CO_2_, which require emission control systems to comply with environmental regulations [68].

#### 2.3.1. Reaction Conditions Screening

Reaction condition screening was performed using 2.00 g of rapeseed oil and 20.0 g of hexane, stirring at 200 rpm, and a fixed time of 6.0 h. The temperature, H_2_ pressure, and catalyst/oil wt% ratio were varied according to the reported values: T = 320 °C, 240 °C, P(H_2_) = 20 bar, 40 bar, catalyst/oil wt% ratio = 10%, 4%. A reaction under intermediate conditions was also carried out (280 °C; 30 bar H_2_ pressure; 7.0 wt% catalysts).

**Figure 5:**
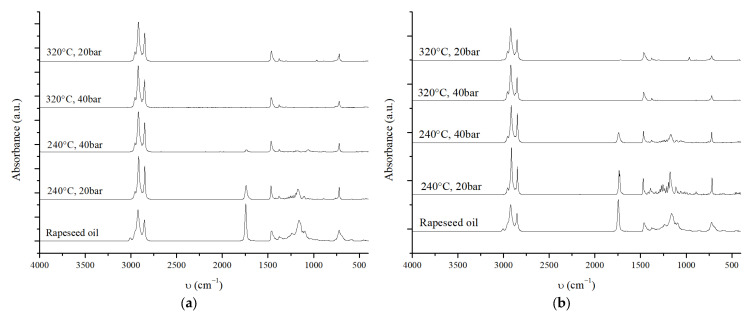
(**a**) IR spectra of the OLP obtained using 10 wt% of NiMoAl R., with reaction conditions specified in the figure); (**b**) IR spectra of the OLP obtained using 4 wt% of NiMoAl R, with reaction conditions specified in the figure. Fixed reaction conditions are 2.0 g of rapeseed oil and 20.0 g of hexane for 6 h. The NiMoAl C. catalyst is reduced in batch at 320 °C, 60 bar of H_2_ for 8 h.

A preliminary qualitative analysis of the OLP was performed using FT-IR ATR to evaluate the complete conversion of TGs and detect the presence of free fatty acids, which would indicate incomplete DCO reactions. The FT-IR ATR spectra of the OLP obtained from the reaction screening are shown in Figure 5.

Infrared (IR) analysis enables a rapid and qualitative assessment of the degree of conversion of triglycerides in the oil. The area between 1745–1700 cm^−1^ corresponds to the vibrational modes of the C=O bond in triglyceride esters (around 1745 cm^−1^) and free fatty acids (around 1710 cm^−1^). Consequently, the absence of signals in this region indicates the complete conversion of triglycerides [70]. In addition, the area between 1350–900 cm^−1^ corresponds to the vibration of the C-O bonds; therefore, it indicates the degree of conversion and the absence of oxygenated compounds in the reaction mixture. Figure 5a shows that, when using 10 wt% of the catalyst at 240 °C, the reaction conversion is incomplete, while, at 320 °C, all TGs are fully converted into hydrocarbons. Moreover, when the reaction is carried out at 20 bar of H_2_ pressure, the IR spectrum reveals a low-intensity peak around 966 cm^−1^, probably related to unsaturated carbon bonds, suggesting incomplete hydrogenation due to the lower H_2_ pressure. Interestingly, increasing the pressure from 20 to 40 bar at 240 °C significantly reduces the carbonyl bond peak, indicating that higher pressure promotes oil conversion.

Using a lower catalyst amount (4 wt%) (Figure 5b), complete conversion is achieved only at 320 °C and 40 bar H_2_, confirming that higher temperatures are required to convert oil fully. As observed for the reaction with a 10 wt% catalyst, a pressure effect is also evident. At lower pressures (20 bar of H_2_), the IR spectrum reveals two weak peaks: one corresponding to the ester signal, indicating incomplete conversion, and the other associated with the double bond signal, suggesting the incomplete hydrogenation of the double bonds into saturated hydrocarbons.

A more detailed analysis of the hydrocarbon composition in the OLP mixture was performed using GC-FID and GC-MS (Table 3).

In all studied cases, to achieve 100% conversion (no FAME content, i.e., no residual oil), the reaction must be carried out at temperatures above 280 °C, as is consistent with the results of the IR analysis. Comparing tests carried out under the same reaction conditions but a different temperature (entries 1 vs. 6, 2 vs. 7, 3 vs. 8, and 4 vs. 9) shows that higher conversion values (usually 100%) and more C_15_-C_18_ linear saturated hydrocarbons (i.e., green diesel hydrocarbons) are observed at higher temperatures. At 240 °C (entries 7–9), a remarkable amount of FAME was obtained due to incomplete conversion. Conversely, at higher temperatures (entries 1–4), small numbers of branched hydrocarbons (1.8–4.5%) and C_8_-C_14_ linear saturated hydrocarbons (1.7–7.0%) were detected as a result of competitive cracking reactions. The positive effect of the temperature on the CDO reaction has been reported by many authors [10,71,72,73]. Additionally, at elevated temperatures, the hexane (i.e., the solvent) reaches a supercritical state, enhancing the hydrogen transport capacity and solubility in hexane, thereby promoting higher conversions [74,75].

H_2_ pressure also positively influences the CDO reaction. In all studied cases (entries 1 vs. 2, 3 vs. 4, 6 vs. 7, and 8 vs. 9), a significant amount of C_15_-C_18_ yield was observed with higher pressure. At 240 °C, where conversion is incomplete (entries 6–9), a remarkable increase in this value is observed as the pressure increases (i.e., from 45 to 94% entries 7 and 6, respectively) [76,77,78,79]. At 320 °C and 20 bar H_2_ pressure (entries 2 and 4), olefins (mainly C_17_ and C_18_) were also detected, probably due to the lower hydrogen concentration in the reaction mixture. In addition, lower pressure slightly enhances the cracking reaction, favoring the formation of *n*-C_8_-C_14_ hydrocarbons (entries 2 and 4) [80]. The effect of pressure can be attributed to the increased solubility of H_2_ at higher pressures.

At higher temperatures, the catalyst amount has a negligible effect on conversion (entries 1 vs. 3; 2 vs. 4). However, at lower temperatures (240 °C), increasing the catalyst amount significantly enhances conversion and the C_15_-C_18_ percentage (see entries 6 vs. 8; 7 vs. 9). For example, at 240 °C and 40 bar H_2_ pressure, conversion rises from 37 to 94%, and the C_15_-C_18_ yield increases from 30.3 to 88.2% (entries 6 and 8). At both 4 wt% and 10 wt% of the catalyst, the best reaction conditions are 320 °C and 40 bar, resulting, in both cases, in a 100% hydrocarbon biofuel, of which 94% (entry 1) and 92% (entry 3) is in the diesel range, and 65 wt% and 67 wt% is the green diesel yield, respectively.

Focusing on the OLP yield, as conversion increases, the OLP yield decreases because more gaseous products are formed, including propane (deriving from the hydrodeoxygenation of the glycerol of the triglyceride), CO, and CO_2_ (deriving from the DCO and DCO_2_ reactions).

Since the green diesel yield (GD%) is calculated based on both the recovered OLP and the *n*-C_15_-C_18_ content (see Equation (2)), higher GD yields may sometimes be observed in tests with incomplete conversion (see for example entries 1 vs. 6). However, incomplete conversion leads to undesired products, such as fatty acids and unreacted triglycerides, compromising product quality. Temperature and pressure also influence reaction selectivity (S). In our case, higher temperatures seem to promote the HDO reaction, i.e., higher C_16_ and C_18_ hydrocarbon content (see Equation (3)). Given the exothermic nature of the HDO reaction, the reverse would be expected, but this can be explained by the fact that the hydrogen-shuttling capacity of supercritical hexane improves hydrogen solubility, promoting HDO selectivity [74,75]. Increased pressure (working at 320 °C) also results in higher S values due to the significant hydrogen consumption presented by this reaction pathway. Finally, the catalyst % does not affect the reaction selectivity. It should be noted that the S value does not influence the quality of the product obtained in any measure, giving only a suggestion about the reaction pathway.

A test under intermediate reaction conditions (entry 5) was also performed to confirm the effect of varying reaction conditions on the overall reaction. In this case, incomplete conversion (97%) and lower diesel-range hydrocarbon content (86.4%) are obtained.

Based on the above findings, the best reaction conditions in terms of achieving the best compromise between the OLP yield, GD yield, and C_15_-C_18_ content are those reported in entry 3 (T = 320 °C; P(H_2_)= 40 bar; 4 wt% catalyst; reaction time 6 h).

#### 2.3.2. Reaction Time Effect

**Figure 6:**
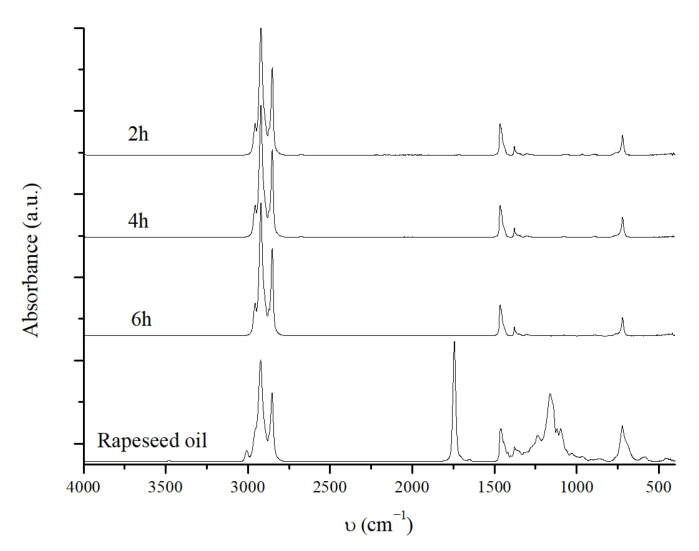
IR spectra of the OLP obtained at different reaction times and with 4 wt% of NiMoAl R., 320 °C, 40 bar H_2_, 2.0 g of rapeseed oil, and 20.0 g of hexane. The NiMoAl C. catalyst is reduced in batches at 320 °C, 60 bar of H_2_, and for 8 h.

The effect of the reaction time was also investigated, by referring to the best reaction conditions in Table 3. Two further reactions were conducted using the conditions reported in entry 3 of Table 3, with reaction times of 2 h and 4 h. A preliminary IR analysis of the products obtained at 6, 4, and 2 h (Figure 6) did not seem to show any difference.

In all cases, signals related to C=O and C-O bonds are absent, suggesting complete conversion even at a reaction time of 2 h.

Moreover, GC-FID and GC-MS analyses show no significant differences between reactions performed at different times; 100% conversion and *n*-C_15_-C_18_ hydrocarbon content higher than 90% are always obtained (Table 4).

Slight differences are observed for the reaction performed for 2 h, in which very small amounts of methyl stearate (0.4%) and other compounds (1.1%) are detected (entry 1, Table 4 footnote 2). However, given the negligible percentages of these compounds, it can be confirmed that the NiMoAl catalyst effectively converts rapeseed oil even after only 2 h of reaction time, producing a hydrocarbon biofuel consisting of 91% hydrocarbon in the diesel range and with a green diesel yield of 73.6 wt%. In addition, it is observed that shorter reaction times result in higher OLP yields, 72.9 wt% after 6 h (entry 3) vs. 81.2 wt% after 2 h (entry 1); this should not be surprising, since a shorter reaction time also means less time for cracking reactions to take place. Additionally, a slight decrease in selectivity is noted as the reaction time decreases, starting from an S value of 2.7 at 6 h, which is reduced to 2.2 after 2 h of reaction time. It is important to note that reducing the reaction time to 2 h, while keeping the conversion and hydrocarbon distribution almost unchanged and achieving a higher diesel yield, offers a very good result, since a shorter reaction time results in greater energy savings.

#### 2.3.3. The Effect of the Reduction Time of the Catalyst

**Figure 7:**
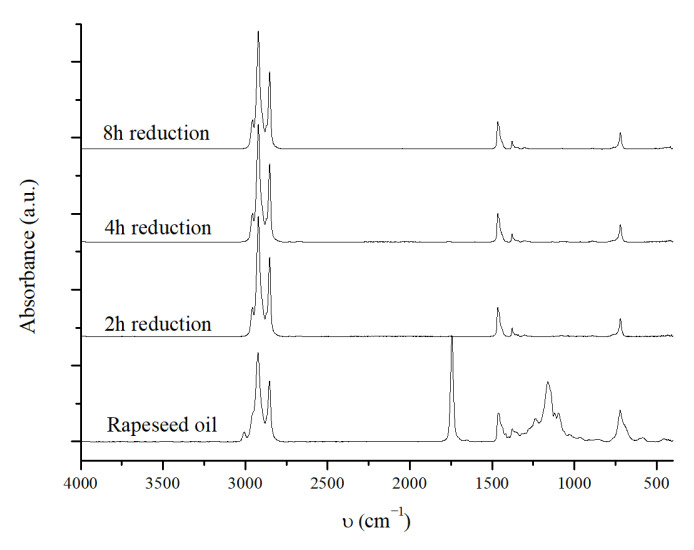
IR spectra of the OLP obtained at 320 °C, 40 bar H_2_, 2 g of rapeseed oil, 20 g of hexane, 6 h of reaction time, and 4 wt% of the NiMoAl R. catalyst activated at different reduction times.

Still considering the best reaction conditions shown in Table 3 entry 3, the effect on the CDO reaction of the reduction time of the catalyst in the pre-reduction step was also evaluated. For this purpose, the catalyst was reduced for 2 h or 4 h at 320 °C and 60 bar of H_2_ and then tested at 320 °C, 40 bar of H_2_, 4 wt% of catalyst, 2 g of rapeseed oil, 20 g of hexane, and for 6 h. No significant differences were observed from the IR spectra of the reaction mixture obtained at different catalyst reduction times (Figure 7). This evidence indicates that the catalyst can fully convert the oil after only 2 h of reduction.

The GC analysis (Table 5) confirms the IR results.

In any case, there is no effect due to the reduction time, and the hydrocarbon distribution is essentially the same, with diesel-range hydrocarbon content greater than 90%. The differences are more pronounced when we focus on the reaction selectivity and OLP yield. As the reduction time decreases, a decrease in HDO selectivity and an increase in the OLP yield is observed; from entry 3 to entry 1, the OLP yield increases from 72.9 wt% to 81.1 wt% and, since the hydrocarbon distribution is the same, the green diesel yield increases (from 67.1 wt% to 75.5 wt%). These trends can be attributed to the lower degree of reduction of Mo and Ni. Mo-based catalysts are known to promote HDO reactions, so a lower amount of reduced Mo could probably decrease HDO selectivity; with the reduction condition used in this work, the most probable state of reduced Mo is MoO_2_. Simultaneously, the presence of lower amounts of metallic Ni could decrease cracking activity, resulting in a higher OLP yield [31]. It is important to note that the reduction of the catalyst is essential for a positive outcome of the reaction and, based on our results, a reduction time of two hours is sufficient to obtain very good results in terms of conversion and the diesel yield, and this results in significant time and energy savings.

#### 2.3.4. NiMoAl (0.6) R. Recycling Tests

**Figure 8:**
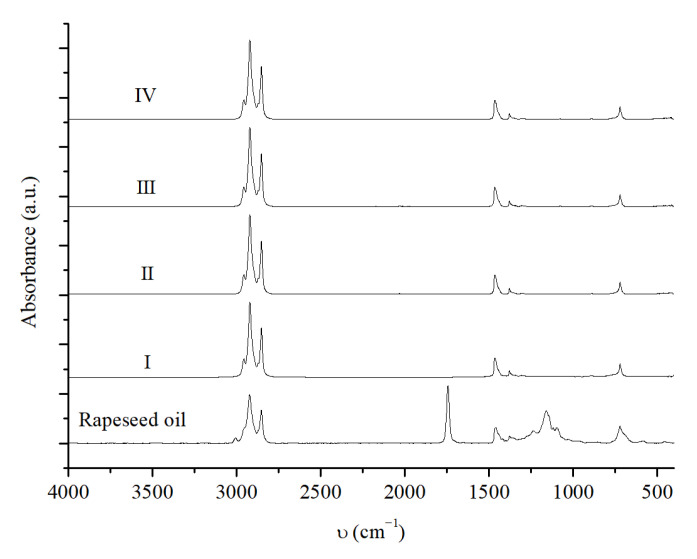
IR spectra of the OLP obtained from successive reaction cycles using 10 wt% of the NiMoAl R. catalyst, 320 °C, 40 bar H_2_, and 2 h of reaction time. The catalyst is pre-reduced only prior to the first reaction at 320 °C, at 60 bar of H_2_ and for 4 h. Roman numerals refer to recycling tests reported in Table 6.

Finally, catalyst activity was investigated for multiple reaction cycles; after each reaction, the catalyst was recovered and dried overnight in a vacuum oven. Once dry, it was reused for the subsequent reaction without further treatment. The reaction conditions were set at 320 °C, 40 bar H_2_, 2 h reaction time, and 10 wt% catalyst (10 wt% of catalyst was chosen to ensure that there was sufficient cycle reaction after each catalyst recovery). The catalyst was reduced only prior to the first reaction at 320 °C, 60 bar of H_2,_ and for 4 h. In the first reaction, 2 g of rapeseed oil was used, and, for the subsequent reaction cycles, the amount of starting oil was adjusted according to the recovered catalyst weight (to maintain the 10 wt% catalyst-to-oil ratio). Similarly, the solvent amount was adjusted to maintain the oil-to-hexane ratio at 1:10. The results obtained from the IR analysis of the reaction mixture are shown in Figure 8.

A comparison of the IR spectra indicates that, at least up to the fourth reaction cycle, the catalyst is able to fully convert the oil to hydrocarbons without any appreciable change in the biofuels produced. For each cycle, no signals corresponding to triglyceride esters are observed, and the spectra show signals related only to C-H and C-C bonds.

The GC analysis reported in Table 6 confirms the observations of the IR analysis.

Even at the fourth reaction cycle, the catalyst remains active, leading to 100% conversion and an almost unchanged hydrocarbon distribution for each reaction cycle. For each reaction cycle, the diesel-range hydrocarbon content exceeds 90%, and low cracking activity is observed. Reaction selectivity slightly changes, but the HDO reaction is always prevalent. Finally, the OLP yield and the green diesel yield remain almost unchanged.

#### 2.3.5. Catalyst Activity Without the Pre-Reduction Step

**Figure 9:**
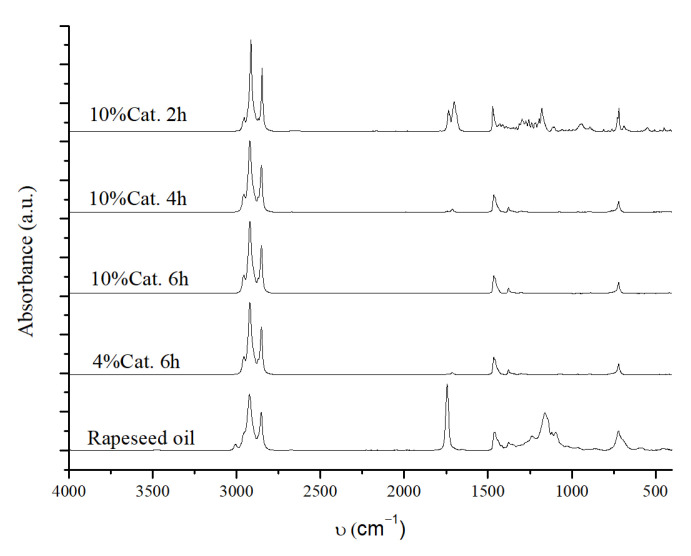
IR spectra of the OLP obtained using the NiMoAl catalyst in the CDO reaction in its oxide form NiMoAl C. (i.e., without the previous activation by reduction). The fixed reaction conditions are: 320 °C, 40 bar H_2_, 2 g of rapeseed oil, and 20 g of hexane.

The catalytic activity of NiMoAl is also evaluated without the previous reduction step, using the catalyst directly in its oxidized form (NiMoAl C.). The activity of NiMoAl C. is evaluated by fixing the reaction conditions at 320 °C, 40 bar H_2_, 2 g of rapeseed oil, and 20 g hexane while the catalyst percentages (10 wt% and 4 wt%) and reaction time (6 h, 4 h, and 2 h) are varied. The IR spectra of the reaction mixture obtained using NiMoAl C. are reported in Figure 9.

The IR analysis shows a low-intensity signal corresponding to the carbonyl group for the reaction performed for 6 h and 4 wt% catalyst, which is absent when using a 10 wt% catalyst. This indicates that, without the reduction activation step, a higher catalyst loading is required for complete conversion. Considering the effect of the reaction time, triglyceride signals are evident at 4 h and particularly at 2 h. In addition, after 2 h reaction time, two peaks related to the carbonyl group are present, one related to the ester carbonyl of triglycerides and one characteristic of the carbonyl of free fatty acids. This suggests that more time is needed to convert both triglycerides and free fatty acids into hydrocarbons under these conditions. The GC-FID results are summarized in Table 7.

With 4 wt% of the catalyst, even after 6 h (entry 4), the reaction mixture contains a small amount of methyl stearate (2.4%). In contrast, when using 10 wt% of the catalyst (entry 3), the biofuel consists of 100% hydrocarbons, and 100% conversion is achieved. At complete conversion, comparing Table 7 entry 3 with the analogous case after catalyst reduction (Table 3 entry 1), it can be seen that a slight decrease in diesel-range hydrocarbons (87.3% vs. 94.1%) and the green diesel yield (60.6% vs. 65.5%) is obtained for the reaction performed without the previous reduction step. However, considering the significant savings in time and cost that are achieved using the calcined catalyst directly, this result is promising. For shorter reaction times (Table 7; entries 1 and 2), the presence of methyl esters at 4 h and 2 h is observed (3.2% and 32.6%, respectively). In this case, it is interesting to note a considerable increase in conversion by varying the reaction time from 2 h (72% conversion, entry 1) to 4 h (98% conversion, entry 2). Regarding selectivity, the oxidized catalyst shows a reversed trend compared to the reduced catalyst, favoring the DCO–DCO_2_ pathway over HDO. This could be explained by the fact that, without the previous reduction step, part of the hydrogen in the system is consumed to reduce the catalyst during the reaction; therefore, there is less available to carry out the HDO reaction, which is the reaction pathway which requires the greatest consumption of H_2_. To summarize, the catalyst does not necessarily have to undergo the reduction step; in fact, under the conditions of entry 1 Table 7, we obtain 100% conversion and a fully hydrocarbon biofuel. A similar reaction carried out with the reduced catalyst leads to slightly better yields and more hydrocarbons in the diesel range, but, considering the considerable energy savings (in terms of H_2_ consumption and thermal energy) in carrying out the reaction without the reduction step, we can say that this is the better result.

### 2.4. Summary of Results

The results obtained in this study demonstrate the excellent catalytic performance of the NiMoAl catalyst for the hydrodeoxygenation (HDO) of rapeseed oil, achieving high selectivity towards green diesel hydrocarbons. Under optimized conditions (320 °C, 40 bar H_2_, 4 wt% catalyst), the catalyst provides, in 2 h, complete conversion, producing a hydrocarbon biofuel with a diesel-range hydrocarbon content exceeding 90%. These findings underscore the catalyst’s efficiency in deoxygenating triglycerides, yielding a high-quality green diesel with a significant reduction in reaction time compared to conventional systems. The recyclability tests confirmed the stability of NiMoAl over multiple reaction cycles. The catalyst maintains consistent conversion efficiency and hydrocarbon distribution, showing good resistance to deactivation and no significant loss of activity, thus improving the economic feasibility of the process. Furthermore, the catalyst in its calcined form (NiMoAl C.) showed promising activity, providing selectivity and green diesel yields comparable to the catalyst in the reduced form (NiMoAl R.), although with longer CDO reaction times. This suggests that the pre-reduction step, while beneficial, may not be strictly necessary for achieving high conversion rates, offering an alternative strategy to lower operational costs. The ability to avoid the reduction step could provide advantages in large-scale applications, where minimizing pre-treatment steps can lead to significant time and cost savings.

It is not easy to compare the results of the catalyst used in this work with other catalysts reported in the literature for the CDO reaction due to the different substrates used, the different processes (i.e., batch vs. flow processes) and analytical techniques used for the characterization of the product, and the different metric used. In principle, we can confirm that they are in line with or sometimes better than the results present in the literature. Our catalyst presents several advantages relative to the classical classes of catalysts used for CDO reactions. Sulfided catalysts are widely used [17,21,71,81], and they show very good activity at high temperatures and pressures. However, they suffer some drawbacks, such as the sulfur contamination of the product due to sulfur leaching from the catalyst. However, they are particularly susceptible to poisoning by alkalis and metals that could be present in the feedstock, and, to maintain activity, they require the continuous addition of sulfiding agents (typically dimethyl disulfide (DMDS), CS_2_, and H_2_S) that are toxic and contaminate the final product, which is undesirable for fuel applications [23,82,83,84,85]. Nitride and carbide catalysts have been explored as sulfur-free alternatives for the deoxygenation of vegetable oils. While these catalysts exhibit promising activity, their synthesis requires severe reaction conditions with an activation process ranging between 700 and 900 °C [41,42,44,86,87,88]. Noble metal-based catalysts, such as Pd, Pt, and Ru supported on various oxides, have also been employed for vegetable oil HDO, demonstrating excellent activity and selectivity [89,90,91]. They achieve complete conversion at lower temperatures [24], but the high cost and limited availability of noble metals hinder their large-scale application.

Overall, the NiMoAl catalyst stands out as an efficient and economically viable system for rapeseed oil hydrodeoxygenation. Its ability to achieve full conversion in shorter reaction times and without the pre-reduction step, combined with the absence of sulfur contamination, lower costs compared to noble metals, and easier synthesis compared to nitrided/carbided systems, positions it as a promising alternative to actual CDO catalysts.

## 3. Materials and Methods

### 3.1. Catalyst Synthesis

The NiAl LDH precursor was synthesized using the co-precipitation method proposed by Santiago Arias et al. [60]. The quantities were calculated to synthesize an LDH with the formula Ni_(1−x)_Al_x_(OH)_2_(C_8_H_4_O_4_)_x/2_∙*n*H_2_O (Ni/Al molar ratio = 0.65, x = 0.6). Briefly, 400 mL of H_2_O milli-Q was boiled for 1 h under continuous Ar flow to prevent carbonate formation in the LDH layers. At 50 °C, under constant stirring and while maintaining the pH in the range of 6.3–6.8, two aqueous solutions (H_2_O milli-Q) were added dropwise in the previous boiled water. The first solution (100 mL) contained 0.020 moles of Ni(NO_3_)_2_∙6H_2_O (98%, Alfa Aesar, Haverhill, MA, USA) and 0.031 moles of Al(NO_3_)_3_∙9H_2_O (≥98% Sigma-Aldrich, St. Louis, MO, USA). The second solution (100 mL) contained 0.017 moles of C_8_H_4_O_4_ (Fluorochem, Glossop, UK) and 0.101 moles of NaOH (pearl, 97%, Lancaster, Ward Hill, MA, USA) as a precipitating agent. After the addition, the mixture was kept at 50 °C for 4 h and then aged for 16 h at room temperature. The resulting solid was filtered under vacuum with H_2_O milli-Q until neutral and subsequently dried in an oven at 110 °C for 4 h. This solid is named NiAl LDH.

Molybdenum was introduced via ion exchange between the terephthalate and the heptamolybdate anion [61]. Then, 1 g of the dried NiAl LDH precursor was placed in a Teflon beaker with a 0.07 M solution of (NH_4_)_6_Mo_7_O_24_∙4H_2_O (99%, Alfa Aesar, Haverhill, MA, USA) in H_2_O mill-Q (the amount of heptamolybdate was set to 50% excess with respect the stoichiometric amount required). The Teflon beaker was then placed in a stainless-steel reactor, and the ion exchange was carried out under hydrothermal conditions at 80 °C with continuous stirring for 24 h. Finally, the resulting solid was filtered under vacuum, washed with H_2_O milli-Q until neutral, and dried at 110 °C in an oven for 4 h. At this stage, the solid was designated as NiMoAl LDH.

NiMoAl mixed oxides (named NiMoAl C.) were obtained via calcination under air at 450 °C (10 °C/min) for 3 h.

Finally, the catalyst was reduced in batch at 320 °C, 60 bar H_2_ and for 8 h. The final form of the catalyst is labeled NiMoAl R.

### 3.2. Catalyst Characterization

The elemental composition of the studied catalyst was performed using an iCAP TQe ICP-MS (Thermo Scientific, Waltham, MA, USA) equipped with a triple quadrupole MS detector. In brief, 20 mg of the solid was dissolved in a concentrated strong acid solution (suprapure nitric acid, 65%, Merck KgaA, Darmstadt, Germany) and then diluted in 200 mL of H_2_O milli-Q. The dissolution process was facilitated by sonication for 10 min. Once dissolved, 10 μL of this solution was further diluted in 20 mL of H_2_O Milli-Q containing 300 μL of 65 wt% HNO_3_ and 200 μL of the internal standard (indium). An aliquot of the final solution was transferred to a test tube for analysis, and the data were processed using QTEGRA software (version 2.10.4345.2366).

The FT-IR measurements were performed using a PerkinElmer Spectrum instrument operating in ATR mode (PerkinElmer, Shelton, CT, USA), and the IR spectra were analyzed using Spectrum software (version 10.7.2). The ATR crystal is made of diamond, with an available area of a 2 × 2 mm. Prior to analysis, the sample was finely ground to enhance the quality of the acquired spectrum. The IR spectrum was obtained by averaging 16 scans with 4 cm^−1^ resolution.

The phase composition of the catalyst was determined using a PANalytical X’Pert PRO diffractometer (Malvern Panalytical, Malvern, UK) with Bragg–Brentano geometry that uses Cu Kα1 radiation (1.540598 Å). Diffractograms were recorded over a 2θ angle ranging from 5° to 90° and analyzed using X’Pert HighScore Plus collector software(version 4.9). The powder sample was ground and sieved with standard sieves (UNI 2331 and ISO R565) [92,93] to obtain a powder with dimensions less than 150 μm. The prepared sample was pressed onto a zero-background sample holder. Phase identification was performed by matching the obtained diffractograms with an international reference database (inorganic crystal structure database (ICSD)).

The textural properties of the catalyst, including the surface area, pore volume, pore diameter, and pore size distribution, were determined via N_2_ physisorption using a NOVA 1200e Surface Area and Pore Size Analyzer (Quantachrome Instruments, Boynton Beach, FL, USA). BET-BJH method calculations were performed using Nova Station A software (version 11.0). The sample under investigation (100–200 mg) was degassed under high vacuum at 200 °C (10 °C/min) for at least 8 h before the physisorption analysis was performed.

### 3.3. Catalytic Deoxygenation Reaction and Product Analysis

The catalytic deoxygenation reactions were carried out in a 100 mL stainless-steel batch reactor (Parr batch reactor 4590 Micro Bench Top Reactors, Parr Instrument Company, Moline, IL, USA). Before the reaction, the required amount of catalyst was reduced in the autoclave at 320 °C, 60 bar H_2_, for 8 h. After the reduction step, the reactor was charged with 2.0 g of rapeseed oil, 20 g of hexane (HPLC grade, ≥95%, Sigma-Aldrich), and 0.200 g (catalyst/oil wt% = 10%) or 0.08 g of the catalyst (catalyst/oil wt% = 10%). Before heating, the reactor was flushed three times with N_2_ and three times with H_2_ to ensure an air-free atmosphere. The reactor was then pressurized to the desired H_2_ pressure (40 or 20 bar), the temperature was set to the chosen value (240 or 320 °C), and stirring took place at 200 rpm (kept constant for each reaction). Once the set temperature had been reached, the reaction was carried out for the chosen time. Once the reaction was completed, the reactor was cooled down to room temperature and washed with hexane and CHCl_3_ to recover all the product.

Catalyst recovery and product isolation were performed as follows. The catalyst was vacuum filtered and washed several times with hexane and CHCl_3_ to recover all the reaction mixture. The filtered catalyst was then dried under vacuum at 60 °C overnight. The reaction mixture was collected in a pre-weighed flask, evaporated using a rotary evaporator, dried under vacuum for 30 min, and re-weighed to determine the yield of the organic liquid product (OLP).

A small amount of OLP was analyzed via FT-IR ATR (4 scans, 4 cm^−1^ resolution) to qualitatively assess triglyceride conversion by analyzing the peak at 1745 cm^−1^ corresponding to the C=O stretching of the carbonyl ester. The FT-IR ATR apparatus is the same as that described for the catalyst’s characterization.

**Figure 10:**
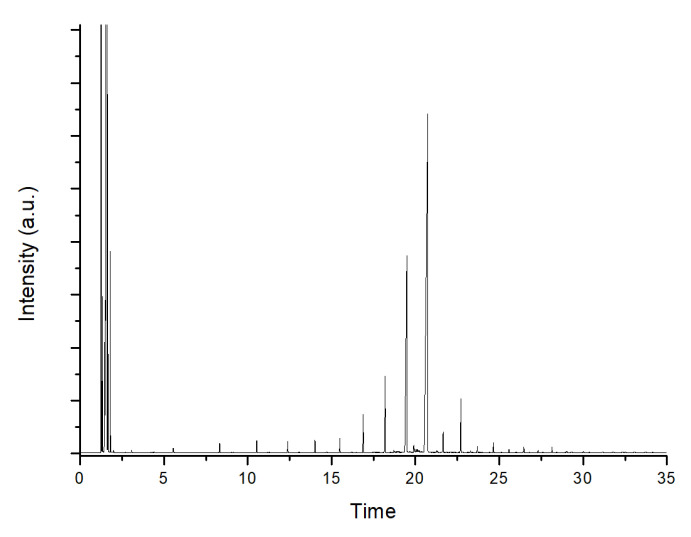
Typical chromatogram of OLP obtained from CDO.

To assess the conversion and distribution of the products in the mixture, a small portion of OLP undergoes transesterification to convert any residual oil into fatty acid methyl esters (FAMEs), which can be easily analyzed using a gas chromatograph. The transesterification reaction was carried out according to the standard AOAC 969.33 [65]. In brief, 1 μL of the transesterified mixture was injected into an Agilent GC/7820A gas chromatograph equipped with a flame ionization detector (FID) and an HP-5 19091J-413 capillary column (30 m × 0.32 mm × 0.25 μm, stationary phase = (5%-Phenyl)-methylpolysiloxane) (Agilent Technologies, Santa Clara, CA, USA). The chromatographic run lasted 35 min, starting at 50 °C (held for 5 min), followed by a temperature increase of 10 °C/min to 280 °C, which was maintained for 7 min. The carrier gas is H_2_, and the injector and FID are set at 250 °C and 300 °C, respectively. Peaks corresponding to *n*-alkanes were identified by a comparison with the C_7_-C_40_ saturated alkane standard (C_7_–C_40_ Saturated Alkanes Standard, 1000 μg/mL each component, Supelco, Bellefonte, PA, USA). Chromatograms were interpreted using OpenLab CDS ChemStation Edition software (version B.01.04.232). In Figure 10, a typical chromatogram of OLP obtained from the CDO reaction is reported.

Additionally, 1 μL of the transesterified sample was analyzed using a Trace GC Ultra 7820 gas chromatograph equipped with a flame-ionization detector (FID) and with a Supelco^®^ SP-2380 GC column (30 m × 0.25 mm × 25 µm) to identify different FAME isomers if present (Agilent Technologies, Santa Clara, CA, USA). The analysis was performed under isothermal conditions (H_2_ carrier; injector 250 °C; column 180 °C for 25 min; FID 250 °C). FAME identification was achieved by conducting a comparison with commercially available standards (FAME MIX, C_14_-C_22_, Supelco), and the chromatograms were processed using Xcalibur software (version 1.0.2.65.SP2).

Any unidentified chromatographic peaks were identified after the injection of the transesterified mixture (20 μL of the transesterified sample is diluted in 1.3 mL of hexane) into a GC/MS (GC model: Varian star 3400 cx, MS model: Varian saturn 2000). The GC is equipped with an HP-5 column (30 m × 0.25 µm × 0.25 mm stationary phase = 5% phenyl-methyl polysiloxane). The run had a duration of 35 min, during which the column was initially maintained at 50 °C for 5 min; then, the temperature was increased by 5 °C/min to 280 °C and finally maintained at 280 °C for 7 min. The carrier gas is He (split flow: 50 mL/min). The trap and injector temperatures were both set at 250 °C. *n*-alkanes were identified by performing a comparison with the C_7_-C_40_ standard (C_7_-C_40_ saturated alkanes standard, 1000 μmL/mL eachcomponent, Supelco). GC-MS chromatograms were processed using Varian WS workstation software (version 6.9), and unknown peaks were identified by comparing the corresponding mass spectra with a library (NIST). In addition, the Kovats retention index was also calculated for further validation of the unknown compound [94].

## 4. Conclusions

The NiMoAl catalyst proposed by Arias et al. [60,61] was successfully synthesized, as confirmed by the characterization of the material via FT-IR, XRD, ICP-MS, and N_2_ Physisorption analyses. In this study, the catalyst activity was explored, for the first time, through the catalytic deoxygenation of rapeseed oil, demonstrating high activity for green diesel production. The reaction parameters, including temperature, pressure, and catalyst loading, significantly influenced the conversion efficiency and product distribution, with higher temperatures and pressures enhancing the deoxygenation process and promoting selective hydrocarbon production. In particular, the catalyst exhibited excellent activity, achieving the complete conversion of rapeseed oil under optimal conditions (320 °C, 40 bar H_2_, 4 wt% catalyst), with a diesel range hydrocarbon content exceeding 90%. Notably, complete conversion was achieved in as little as 2 h under optimal conditions, producing a hydrocarbon biofuel with 91.0% hydrocarbon content in the diesel range (*n*-C_15_-C_18_) and a 73.6% GD yield, highlighting the efficiency of the system. The reduction step prior to reaction proved essential for optimal catalytic activity, although the calcined form of the catalyst also achieved promising results, offering a cost-effective alternative with slightly reduced green diesel yields. Finally, the NiMoAl catalyst demonstrated robust performance across multiple reaction cycles, maintaining high conversion efficiency and consistent product composition without significant deactivation. These findings underscore the potential of NiMoAl-based catalysts as effective and economically viable solutions for producing green diesel from vegetable oils. The study contributes to advancing sustainable biofuel technologies, providing a pathway to reducing reliance on fossil fuels and mitigating greenhouse gas emissions.

Future work should explore the long-term stability of the catalyst and the use of other feedstocks (especially waste or biorefinery feedstocks). An in-depth study of feedstock–catalyst interactions and a kinetic study are planned in order to explain the mechanism of catalyst action against triglycerides. To gain deeper insights into the reaction pathway, shorter reaction times should be employed to promote reaction intermediates such as unsaturated glycol difatty acid esters, alcohols, aldehydes, ketones, and esters, which are indicative of the type of occurred reaction. Our findings indicate that triglyceride decomposition follows a β-elimination mechanism, as evidenced by the presence of free fatty acids and the absence of glycerol (confirmed via FT-IR) in incomplete reactions [12]. In addition, an in-depth characterization of the catalyst would indicate a clearer correlation between structure and activity. Finally, an investigation of the effect of hydrogen donor solvents (to mitigate H_2_ consumption) will be undertaken to promote its commercial exploitation [95,96].

## Figures and Tables

**Table 1:** Catalyst elemental composition according to ICP-MS analysis.

Sample	Al (wt%)	Ni (wt%)	Mo (wt%)	Ni/Al ^1^ (Exp.)	Ni/Al ^1^ (Theor.)	Ni/Mo ^1^ (Exp.)	Ni/Mo (Theor.)	Experimental Formula	Theoretical Formula
NiMoAl C.	15.4 ± 0.03	21.1 ± 0.2	21.3 ± 0.01	0.63	0.63	1.63	0.57	Ni_16_Al_26_Mo_10_O_85_	Ni_13_Al_20_Mo_22_O_109_

^1^ Molar ratio.

**Table 2:** Textural properties of the synthesized materials.

Sample	BET ^1^ (m^2^/g)	BJH ^2^ (cm^3^/g)	Average Pore Diameter ^3^ (nm)
NiAl LDH	130	0.43	13.2
NiMoAl LDH C.	120	0.44	14.5
NiMoAl LDH R.	122	0.37	12.2

^1^ refers to surface area; ^2^ refers to pore volume; ^3^ values refer to 4 V/S_BET_.

**Table 3:** Reaction condition screening (fixed reaction conditions were 2 g of rapeseed oil, 20 g of hexane, and 6 h reaction time).

Entry	Reaction Conditions (T °C/Pbar/wt% Cat)	Olefines (%)	*n*-C_8_-C_14_ (%)	*n*-C_15_-C_18_ (%)	>C_18_ (%)	Branch (%)	FAME (%)	Other (%)	S	χ (%)	OLP Yield (wt%)	GD Yield (wt%)
1	320/40/10	--	1.7	94.1	2.0	2.2	--	--	1.8	100	69.1	65.7
2	320/20/10	8.7	7.0	70.5	6.9	4.5	--	2.4	2.4	100	75.7	53.4
3	320/40/4	--	3.1	92.0	2.8	2.1	--	--	2.7	100	72.9	67.1
4	320/20/4	18	6.6	66.5	2.8	1.8	--	4.3	2.3	100	74.7	49.7
5	280/30/7	1.9	3.1	86.4	4.0	0.6	4.0	--	1.3	97	88.3	76.3
6	240/40/10	--	0.1	88.2	2.4	0.1	7.2	2.0	0.7	94	85.6	75.6
7	240/20/10	--	0.2	37.8	0.6	--	57.1	4.3	1.0	45	95.7	36.0
8	240/40/4	0.1	--	30.3	0.8	--	64.3	4.5	0.8	37	97.4	29.5
9	240/20/4	0.1	--	14.6	1.0	--	77.2	7.1	0.8	25	97.1	14.1

**Table 4:** Effect of the reaction time ^1^.

Entry	Reaction Time (h)	*n*-C_8_-C_14_ (%)	*n*-C_15_-C_18_ (%)	>C_18_ (%)	Branch (%)	S	χ (%)	OLP Yield (wt%)	GD Yield (wt%)
1	2.0 ^2^	2.3	91.0	2.9	1.5	2.2	100	81.2	73.6
2	4.0	2.3	93.1	3.2	1.3	2.1	100	76.9	71.6
3	6.0	3.1	92.0	3.8	2.1	2.7	100	72.9	67.1

^1^ The reactions were carried out at 320 °C with 40 bar H_2_ pressure and 4 wt% of catalyst, 2.0 g of rapeseed oil, and 20 g of hexane. The catalyst was previously reduced for 8 h. ^2^ Negligible amounts of methyl stearate (0.4%) and other compounds (1.1%) were detected.

**Table 5:** Effect of the reduction time of the catalyst on CDO reaction ^1^.

Entry	Reduction Time (h)	*n*-C_8_-C_14_ (%)	*n*-C_15_-C_18_ (%)	>C_18_ (%)	Branch (%)	S	χ (%)	OLP Yield (wt%)	GD Yield (wt%)
1	2.0	2.6	92.1	4.1	1.2	1.6	100	81.1	75.5
2	4.0	2.4	93.2	2.8	1.6	2.1	100	76.0	70.8
3	8.0	3.1	92.0	2.8	2.1	2.7	100	72.9	67.1

^1^ The reactions were carried out at 320 °C with 40 bar H_2_ pressure and 4 wt% of catalyst, 2.0 g of rapeseed oil, and 20 g of hexane; reaction time 6.0 h.

**Table 6:** Recycling tests ^1^.

Entry	Cycle	*n*-C_8_-C_14_ (%)	*n*-C_15_-C_18_ (%)	>C_18_ (%)	Branch (%)	S	χ (%)	OLP Yield (wt%)	GD Yield (wt%)
1	I ^1^	1.6	93.7	3.7	1	1.4	100	82	76.6
2	II ^2^	1	91.7	3.3	3.5	2.2	100	83.2	76.0
3	III ^2^	1.1	90.6	3.3	3.3	1.9	100	84.1	76.0
4	IV ^2^	1.1	91.5	3.4	3.4	1.7	100	81.9	74.8

^1^ The reactions were carried out at 320 °C with 40 bar H_2_ pressure and 10 wt% of catalyst, 2.0 g of rapeseed oil, and 20 g oh hexane for 2.0 h. ^2^ An insignificant amount of unsaturated hydrocarbon was found (between 0.5–0.7%).

**Table 7:** Catalytic activity of NiMoAl C. (oxidized state) ^1^.

Entry	Catalyst wt%	Reaction Time (h)	*n*-C_8_-C_14_ (%)	*n*-C_15_-C_18_ (%)	>C_18_ (%)	Branch (%)	S	χ (%)	OLP Yield (wt%)	GD Yield (wt%)
1	10	6	3.1	87.0	3.2	4.2	0.4	100	69.6	60.6
2 ^2^	10	4	4.5	83.6	3.8	2.8	0.3	98.0	76.4	63.9
3 ^3^	10	2	2.8	56.2	2.5	1.6	0.3	72.0	84.7	47.6
4 ^4^	4	6	4.1	86.3	3.6	1.9	0.4	98.0	70.4	60.8

^1^ The fixed reaction conditions were: T = 320 °C, P(H_2_) = 40 bar, 2 g of rapeseed oil, and 20 g of hexane. ^2^ 3.2% of FAMEs were detected. ^3^ 32.6% of FAMEs and 3.2% of other compounds were detected. ^4^ 2.4% of FAMEs and 1.0% of other compounds were detected.

## Data Availability

The original contributions presented in this study are included in the article. Further inquiries can be directed to the corresponding author.
